# Supplementary material for: Identification of Tomato Ve1 Homologous Proteins in Flax and Assessment for Race-Specific Resistance in Two Fiber FlaxCultivars against Verticillium dahliae Race 1
Source: Plants (Basel). 2021 Jan 15;10(1):162. doi: 10.3390/plants10010162 (PMC7830857; doi:10.3390/plants10010162)
Supplement: Supplementary file 1 [file plants-10-00162-s001.pdf]

Supplemental data

Figure S1. Amino acids alignment through MUSCLE (MUltiple Sequence Comparison by Log-Expectation) of the sequences used to generate the phylogram in **Figure 1**. According to BLOSUM62 matrix, the residues in color are the most conserved. Average BLOSUM62 score: low (0.5) in grey, mid (1.5) in turquoise and max (3.0) in blue.

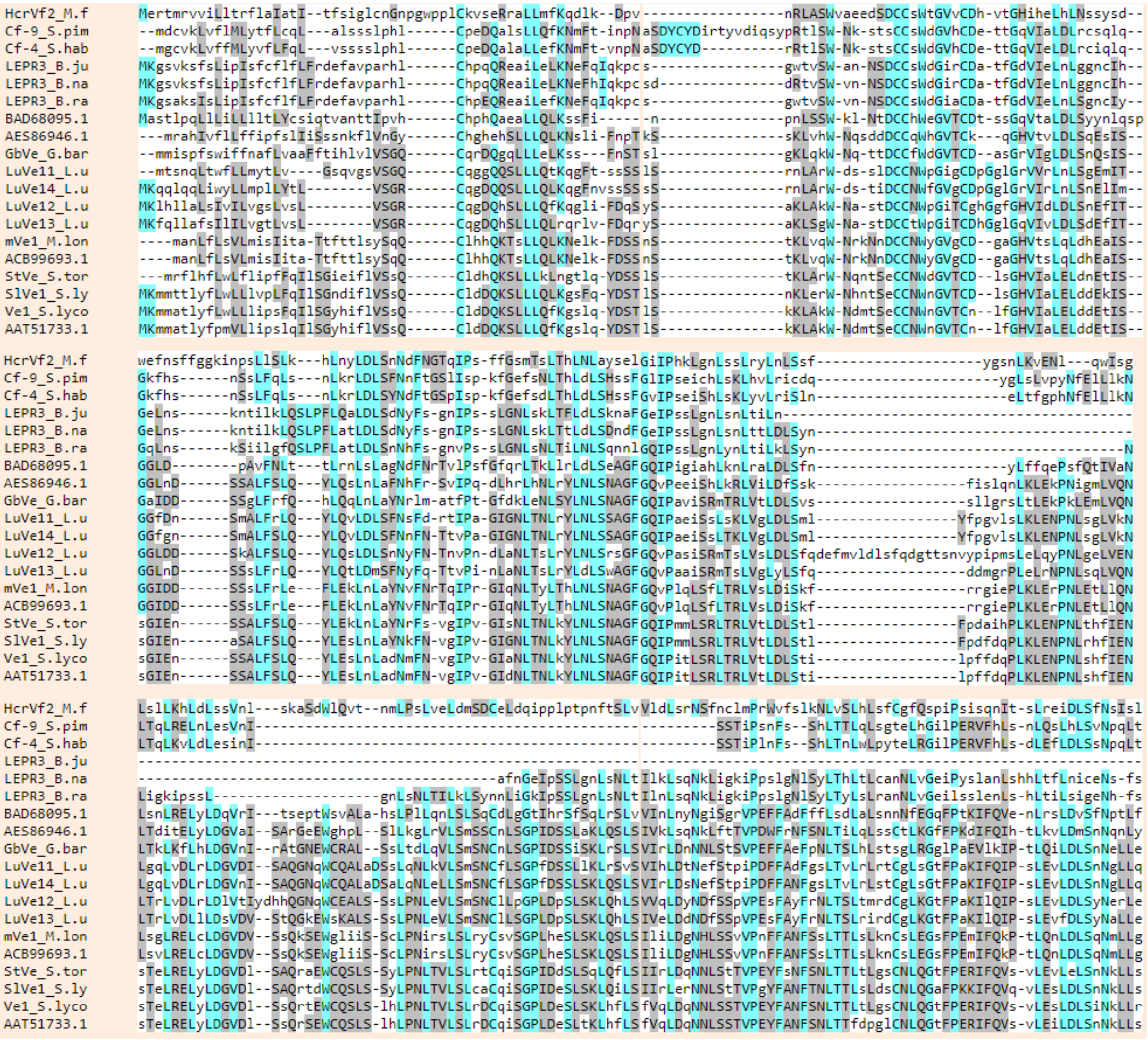

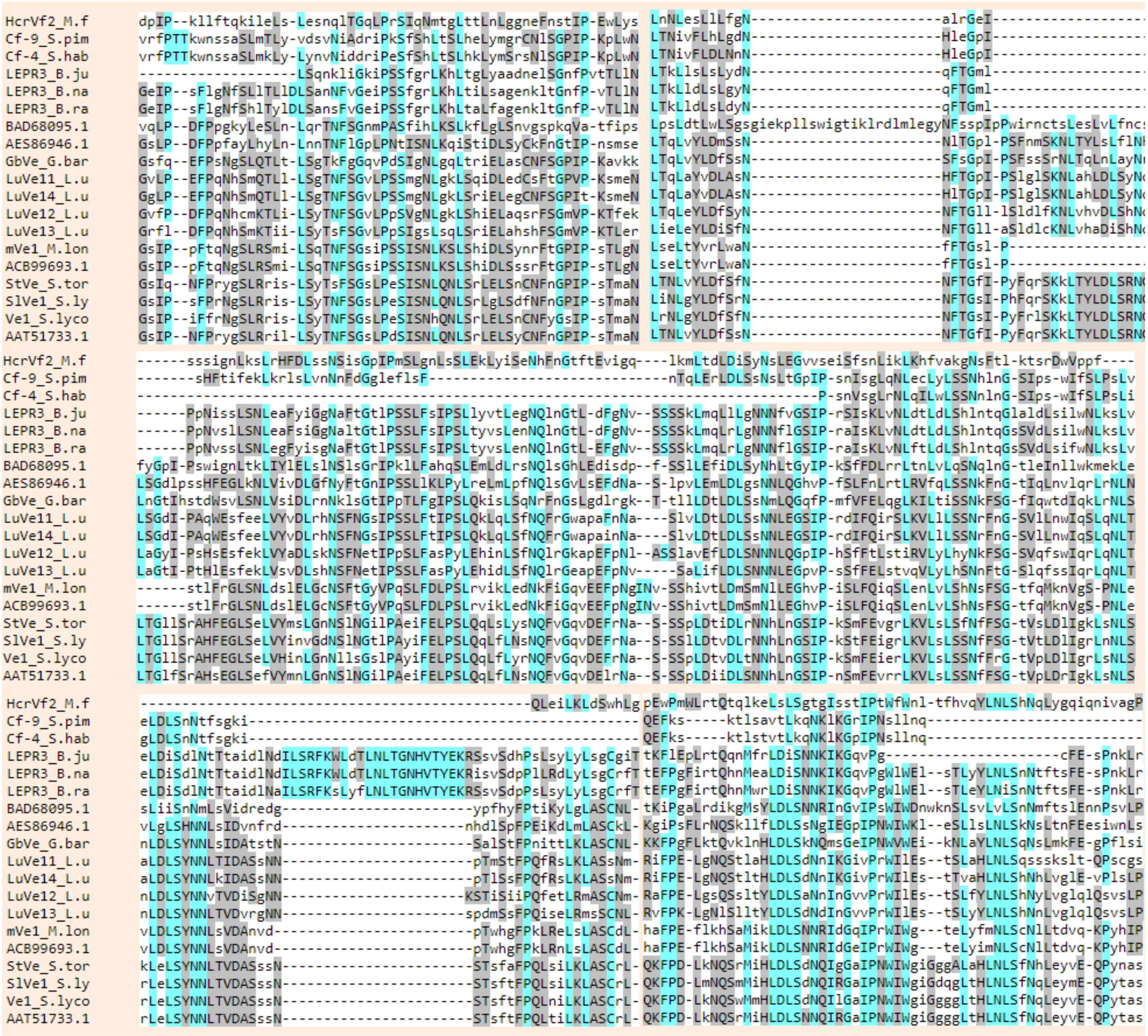

HcrVf2_M.f
Cf-9_S.pim
Cf-4_S.hab
LEPR3_B.ju
LEPR3_B.na
LEPR3_B.ra
BAD68095.1
AES86946.1
GbVe_G.bar
LuVe11_L.u
LuVe14_L.u
LuVe12_L.u
LuVe13_L.u
mVe1_M.lon
ACB99693.1
StVe_S.tor
SlVe1_S.ly
Ve1_S.lyco
AAT51733.1

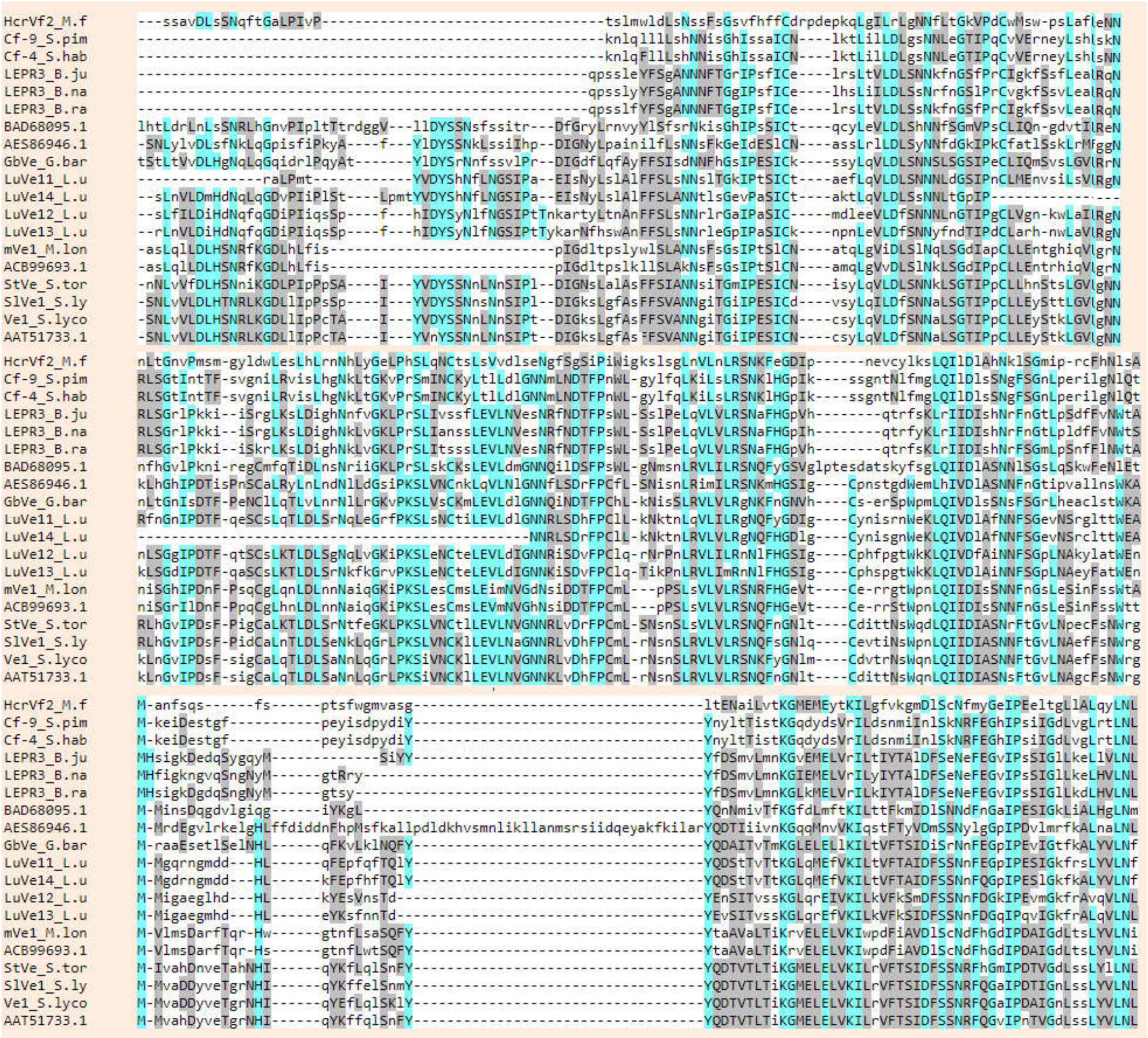
HcrVf2_M.f
Cf-9_S.pim
Cf-4_S.hab
LEPR3_B.ju
LEPR3_B.na
LEPR3_B.ra
BAD68095.1
AES86946.1
GbVe_G.bar
LuVe11_L.u
LuVe14_L.u
LuVe12_L.u
LuVe13_L.u
mVe1_M.lon
ACB99693.1
StVe_S.tor
SlVe1_S.ly
Ve1_S.lyco
AAT51733.1

```
HcrVf2_M.f    SnNrFTGrIPSkiGsmaqLESLDfSmNqLdGEIPpsmtiLTFLshLNLSYNNLtGRIPes tQlQsldqsSFvGN-eLCGaPLNKnCsengvippPtvehdggggyslvedEWfYVslgVG
Cf-9_S.pim    SHNvleGHIPaSFqNLSvLESLDLSsNKiSGEIPQqLASLTFLeVLNLShNhLVGcIPkG kQFdsFgntSyQGNdGLrGfPLsKlCggedqvttPaeldqeeEeEdSpmisWqgVlvgyG
Cf-4_S.hab    SHNvleGHIPaSFqNLSvLESLDLSsNKiSGEIPQqLASLTFLeVLNLShNhLVGcIPkG kQFdsFgntSyQGNdGLrGfPLsKlCggedqvttPaeldqeeEeEdSpmisWqgVlvgyG
LEPR3_B.ju    SgNAFTGHIPSSmGNLSsLESLDLSRNKLtGaIPQEL-------VLhL------------ ------------------------------------------------dsrWdt------
LEPR3_B.na    SgNAFTGrIPSSmGNLSsLESLDLSRNKLtGEIPQELgNLsyLAymNfShNqLVGlvPgG tQFRTqpcsSFKdNPGLfGpsLNqaCvdihgktsqpsemSkeEeEdgqeevisWIaAAIG
LEPR3_B.ra    SgNsFTsHIPSSmGNLSsLESLDLSRNKLtGaIPQELgNLsyLAymNfShNqLVGlvPgG tQFRTqpcsSFKdNPGLyGpsLdqaCvdihgktsqpselSkeEeEdgqeevisWIaAAIG
BAD68095.1    SrNsFTGrIPSkiGkLvqLESLDLSlNqLSeaIPQELASLTsLAILNLSYNNLtGQIPqG pQFlsFgnrSFEGNaGLCGrPLsKqCnysgieaarsp-sSsrD---SvgiiilFVFvgsG
AES86946.1    SHNAlTGHIPSSveNLkhLESmDLSnNsLnGEIPQgLsSLsFLAymNLSFNhLVGRIPlG tQiQTFdvDSFaGNeGLCGpPLtKiCeppqpa------setphsQnesfvEWsFIsieLG
GbVe_G.bar    SHNAFTGsIPpSlGNLSqLESLDLSsNsfdGEIPiqLANLnFisfLNvSnNkLeGQIPrs tQiQsFSeaSFEnNkGLCGlPLttdCvngtspkp----rTtqEfQpAdefDWqFIFigVG
LuVe11_L.u    SHNsFTGpIPSvFGNLSqLESpDLSnNKLtGQIPQELteLTFLsaLNLSdNhLdGRIPtG SQFnTFdnsSFgGNPGLCGsPftKpCsgnst-------tplsErEdesainWnllsiefG
LuVe14_L.u    SHNgFTGeIPStlGNvSqLESLDLSnNKLtGrIPsaLteLTFLsaLNLSYNhLyGRIPtG rQFQTFensSFEGNPnLCGnPftKsCsdiivt------ppaqKesgkpsiDWeFIvpgIG
LuVe12_L.u    SHNAFTGNIPSaFGdLSnmESLDLShNKLkGhIPdELtgmkFLeVLNLSdNNLVGRIPtG NQFQTFdntSFgaNPdLCGiPLtKnCtpsspvhgdat-iShen--hSsrvDWdllsmAfG
LuVe13_L.u    SHNdFTGNIPSaFGdLSnmESLDLShNKLkGhIPEELtgmkFLeVLdLSdNNLVGRIPtG NQFQTFdntSFgGNPdLCGiPLaKnCtpsspvhgdvt-iShen--hSsrvDWevlsmtfG
mVe1_M.lon    SHNAlgGsIPkSlGqLSkLESLDLSRNRLSGhvPtELggLTFLsVLNLSYNeLVGeIPnG rQmhTFSaDaFKGNaGLCGrhLeRnCsdd---------rSqgEiEieneiEWvYVFvALG
ACB99693.1    SHNAlgGsIPeSFGhLSrLESLDLSRNqLtGhvPtELggLTFLsVLNLSYNeLVGeIPnG rQmhTFlaDSFQGNaGLCGrPLeRnCsdd---------rSqgEiEieneiEWvYVFvALG
StVe_S.tor    SyNAleGpIPkSvGkLqmLESLDLStNhLSGEIPsELASLTFLAaLNvSFNNLfGKIPqG iQlQTFSgDSFEGNrGLCGfPLsnsCksdase------lTpapssqddsyDWqFIFkgVG
SlVe1_S.ly    SHNAleGpIPkSiGkLqmLESLDLSRNhLSGEIPsELASLTFLAaLNLSFNkffGKIPst NQFQTFSaDSFEGNsGLCGlPLNdsCqsngses-----lppltsQsdsddEWkFIFAAVG
Ve1_S.lyco    SHNAleGpIPkSiGkLqmLESLDLStNhLSGEIPsELASLTFLAaLNLSFNkLfGKIPst NQFQTFSaDSFEGNsGLCGlPLNnsCqsngsases---lppptplpdsddEWeFIFAAVG
AAT51733.1    SHNAleGpIPkSiGkLqmLESLDLStNhLSGEIPsELASLTFLAaLiLSFNNLfGKIPst NQFlTFSaDSFEGNrGLCGlPLNnsCeskrsefmPlq-tSlpE----sdfEWeFIFAAVG

HcrVf2_M.f    FftGfwivlgsLLvnmpWs-illsqllnRivlkmYhviveyv------------------------------------------------------------------------------
Cf-9_S.pim    cgLviglsViyIMwstqYp-aWF-srmDlkLehiittkmkKhkKry--------------------------------------------------------------------------
Cf-4_S.hab    cgLviglsViyIMwstqYp-aWF-srmDlkLehiittkmkKhkKry--------------------------------------------------------------------------
LEPR3_B.ju    ------------------------------------------------------------------------------------------------------------------------
LEPR3_B.na    FipGiAfgftmeyimvSYKPeWF-----------inlfgrtkrRristtrr---------------------------------------------------------------------
LEPR3_B.ra    FipGiAfgfamgyimvSYKPeWF-----------inlfgrtkrRristtrr---------------------------------------------------------------------
BAD68095.1    F-------------------ktnDlsVqEhmldlqs------------------------------------------------------------------------------------
AES86946.1    FffGfgvfIlPVfcWkklR-lWYskHVDEmLyrFiPrldfvyeqhegkRyktlKwmy---------------------------------------------------------------
GbVe_G.bar    FgVGAAlfVaPLIFWkTas-kWvDeiVDKiLevvlPklg-RtytcpgdRkvdedenlEEdnkgsDeEdeqsqettEeFhGRYCVFCSKLDqTRKkAIHDlsCTCydSSssspsSstsppf
LuVe11_L.u    iiVGfgavtlPaMlWprFR-nWYwprIDsliiavFPqfrshkanatrrRagerRsgr---------------------------------------------------------------
LuVe14_L.u    FgaGAAlaVaPLLFskkan-kWlDdHIDRiLlvllPlfglRyytsndwRrvqpeeilDhyvfydDDEEDEDEsydEfFqGkYCVyCtKLDaTRtmAIHDPKCTCrhSrtgstFSspfsSt
LuVe12_L.u    YvfGlgalmlPaaiWsSFR-iWYwpkVDylLvwmFPklytKdwnnrlvRtrkkKsi--------------------------------------------------ikDdk----piiNh
LuVe13_L.u    YvfGlgalmlPaalWsSFR-iWYwpkVDylLvwmFPklytKdwnnrlvRtqekKsi--------------------------------------------------iNDgk----A-nNy
mVe1_M.lon    YvVGlgiiVwlLLFcrSFR-ykYfdkIDKvvqetFdardrRrrRrgtRivrnqvvrrsh-------------------------------------------------------------
ACB99693.1    YvVGlgiiVwlLLFcrSFR-ykYfdkIDKvvqetFdardrRrrRrgtRivrnqvvrrsh-------------------------------------------------------------
StVe_S.tor    YgVGAAvsIaPLLFYkrgR-kycDkHleRmLklmFPrfgftytRfhpgkvvavehyeDEt---pDDtEDDDEggkEaslGRYCVFCSKLDfqRKeAIHDPKCTChmSSslisFplApsSs
SlVe1_S.ly    YlVGAAntIsPLwFYepvK-kWFDkHaeKwLlwFsrm-----------------------------------------------------------------------------------
Ve1_S.lyco    YiVGAAntIsvVwFYkpvK-kWFDkHmeKcLlwFsrk-----------------------------------------------------------------------------------
AAT51733.1    YiVGAAntIsvVwFYkpvK-kWFDkHmeKcLlwFtrk-----------------------------------------------------------------------------------

HcrVf2_M.f    -----------
Cf-9_S.pim    -----------
Cf-4_S.hab    -----------
LEPR3_B.ju    -----------
LEPR3_B.na    -----------
LEPR3_B.ra    -----------
BAD68095.1    -----------
AES86946.1    -----------
GbVe_G.bar    sp---------
LuVe11_L.u    -----------
LuVe14_L.u    tt---------
LuVe12_L.u    Hm---------
LuVe13_L.u    Hm---------
mVe1_M.lon    -----------
ACB99693.1    -----------
StVe_S.tor    spllvichkkf
SlVe1_S.ly    -----------
Ve1_S.lyco    -----------
AAT51733.1
```

Figure S2. Phylogram showing evolutionary relationships between Ve1 homologs (clade B) including candidates proteins in flax, determined by using maximum-likelihood method. Branch lengths are proportional to evolutionary distances, branch values > 0.6 are given at the node to indicate the branch support. In addition, other Ve1 homologs were added to the tree including HLVe1-2A (*Humus lupulus*; Genebank accession: AIE39594.1); HLVe1-2B (*Humus lupulus*; Genebank accession: AIE39594.1), NgVe1 (*Nicotiana glutinosa*, Genebank accession: ALK26499.1), CaVe (*Capsicum annum* var. *annum,* Genebank accession: AZY92102.1), StuVe1 (*Solanum tuberosum*, Genebank accession: ALK26501.1), StuVe2 (*Solanum tuberosum,* Genebank accession: ALK26503.1), and Ve2 (*Solanum lycopsersicum,* Genebank accession: AAK58012.1). Some of them were tested for *Verticillium* resistance (Song *et al*., 2017), Ve1 homologs providing resistance are indicated in (1) or not in (2).

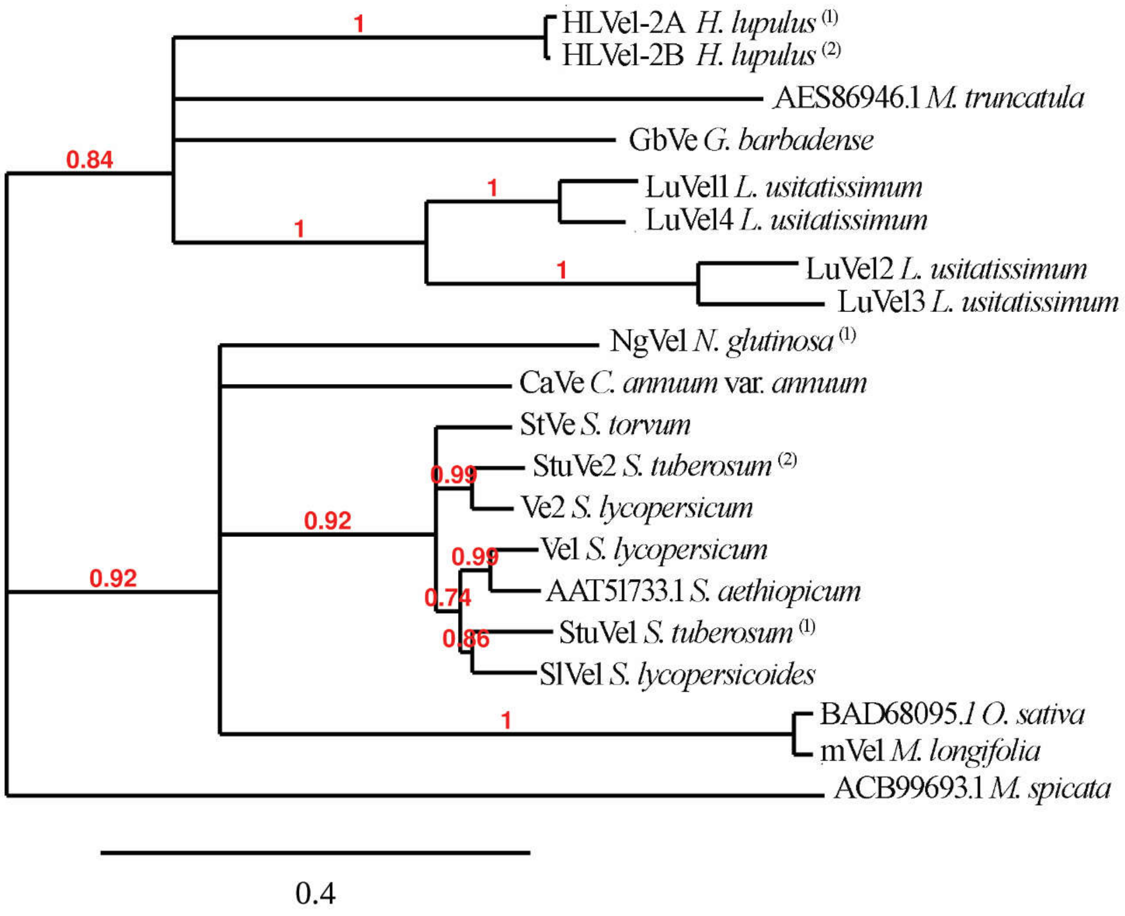

Figure S3. Secondary structure predictions of LuVe11 (up) and Ve1 (down). Colored amino acids indicate structural elements: in grey for coil, in pink for α-helix, in yellow for β-strand and in white for transmembrane segment. Segments B1 to B8 are boxed in green, B20 to B23 in blue and B32 to B37 in red.

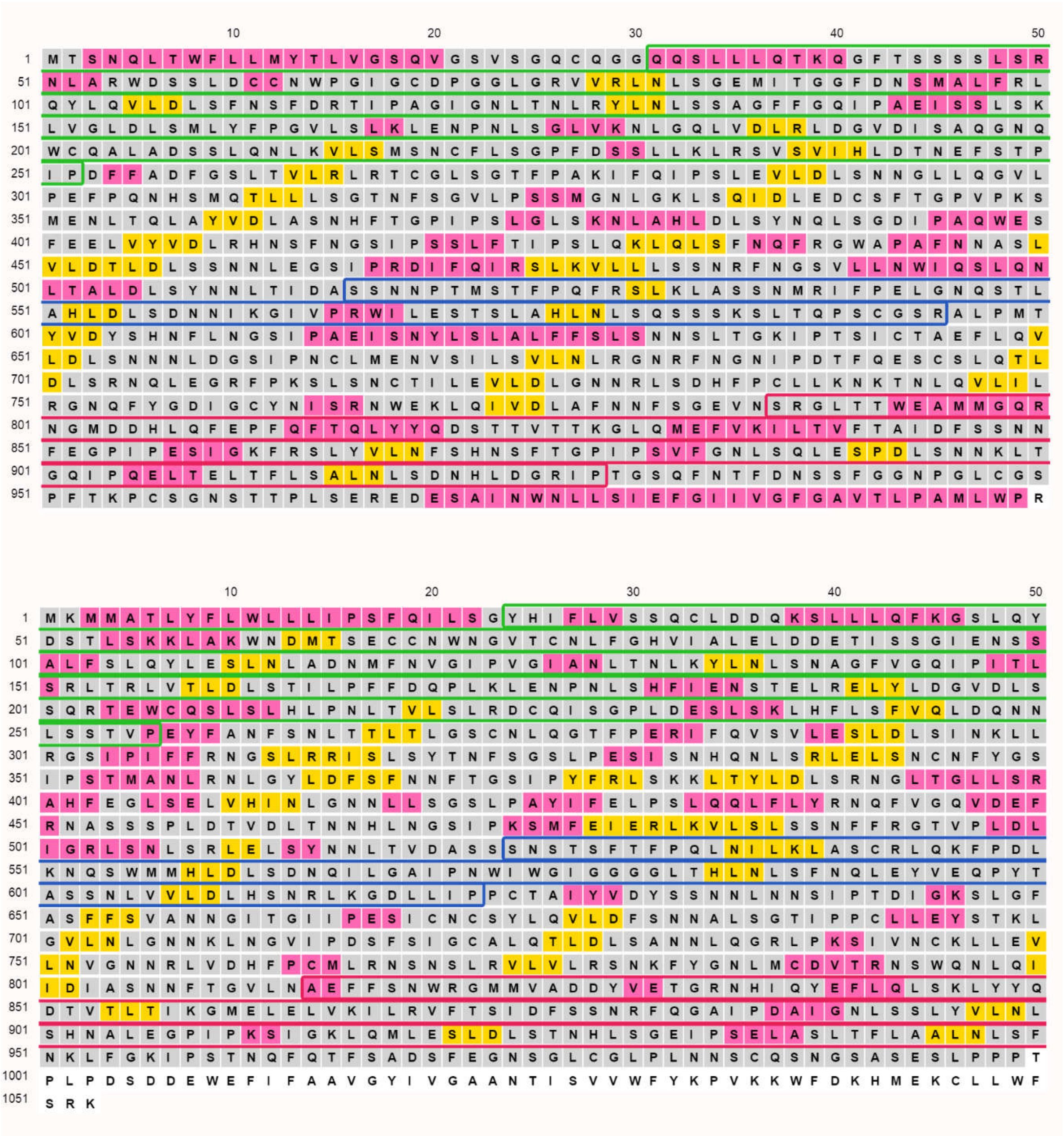

Table S1. Prediction of peptide signal and subcellular localization within the four proteins in flax and Ve1. Values indicate location of each fragment.

Prediction / Sequence	Signal peptide	Non cytoplasmic	Transmembrane	Cytoplasmic
LuVe11	1->25	26->974	975->998	999->1039
LuVe14	1->23	24->933	934->954<br>966->983	955->965<br>984->1061
LuVe12	1->23	24->1016	1017->1038	1039->1090
LuVe13	1->23	24->994	995->1016	1017->1067
Ve1	1->23	24->1010	1011->1032	1033->1053
